# A guide for arterial line insertion for the South African primary care practitioner

**DOI:** 10.4102/safp.v67i1.5976

**Published:** 2025-01-31

**Authors:** Indiran Govender, Henry I. Okonta, Olukayode Adeleke, Sunday Okeke

**Affiliations:** 1 Department of Family Medicine and Primary Health Care, Faculty of Health Sciences, Sefako Makgatho Health Sciences University, Pretoria, South Africa; 2 Department of Family Medicine and Primary Health Care, Faculty of Medicine, Sefako Makgatho Health Sciences University, Pretoria, South Africa; 3 Department of Family Medicine and Rural Health, Faculty of Health Sciences, Walter Sisulu University, Mthatha, South Africa

**Keywords:** arterial pressure monitoring, primary healthcare physician, subclavian vein, resuscitation, intra vascular assess, anatomy

## Abstract

This article provides information on the reasons for inserting an arterial line, how to insert this line, the equipment required to insert an arterial line, and the possible complications that may arise from this procedure. It is hoped this information will guide primary care practitioners working in the South African environment and increase their confidence for inserting arterial lines.

## Introduction

Arterial line insertion is the procedure by which a catheter is placed in the lumen of an artery. It is frequently performed in acute and critical care settings as an invasive means for more accurate direct blood pressure monitoring during titration of vasoactive drugs and intravascular access for blood sampling.^1,2^ The two most common arteries utilised for this procedure are the radial and femoral arteries. The first clinically useful arterial line was successfully developed and placed by Peterson in 1949.^3^

### Contraindications

The absolute contraindications to arterial line insertion include:^1^

- Absent pulse.
- Raynaud’s syndrome.
- Thromboangiitis obliterans (Buerger’s disease).
- Poor perfusion to the extremity.

Relative contraindications are:^1^

- Coagulopathy.
- Atherosclerosis.
- Inadequate collateral flow.
- Infection at the insertion site.
- Previous surgery in the area.

### Equipment

The following equipment are mandatory for arterial line placement:

- Sterile drape.
- Sterile swabs and dressings.
- Two percent chlorhexidine or providone-iodine.
- One percent lignocaine 2 mL ampoule.
- Sterile gloves.
- 4–0 nylon suture or silk 2–0.
- Needle holder.
- Adhesive tape.
- Arterial line flush bag.
- Cannula – this is usually a polyurethane or polyethylene catheter. The size and depth of the artery to be cannulated determines the size and length of the catheter inserted. Shorter and smaller catheters (20–22 gauge, 25 mm – 50 mm) are used for the arteries in the hands and feet, while longer and ticker catheters (14–20 gauge, 15 cm – 20 cm) are used for larger arteries.^2^

The optional equipment include:

- No. 11 blade scalpel.
- Pressure transducer kit.
- A 5 mL syringe.
- 21G needle.
- Hypodermic needle 25G.
- 3-way stopcock.
- Arm board (for placement in radial artery).
- Ultrasound with a sterile ultrasound probe cover.

## Essential anatomy

### Radial artery

**FIGURE 1:**
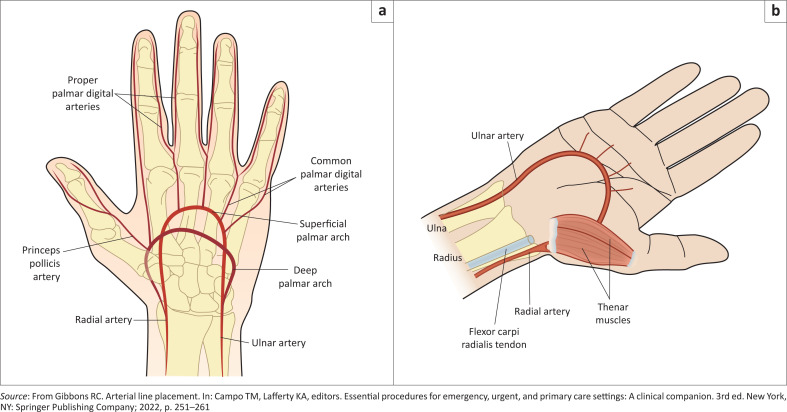
(a) Arterial blood supply of the hand. (b) Radial artery anatomical landmark.

Blood supply to the hand is from the radial and ulnar arteries, branches of which meet in the palm to form the superficial and deep palmar arches (Figure 1a).^4^

The radial artery runs along the lateral aspect of the anterior forearm and can be easily palpated between the styloid process of the radius and the flexor carpi radialis tendon (Figure 1b).

Anatomical variations in the arterial pattern of the upper limb are common and clinically significant. Variations in the origin, course, branches and length of the arterial supply to the hand, particularly in the radial artery, have been documented.^5^ The ulnar artery is typically larger than the radial artery, although in some individuals, it may be of the same size or smaller.^6^ Palmar anastomoses may also vary among individuals. Some may have duplicate palmar arches, while in others, the deep palmar arch may not have any connections to the ulnar artery.^6^ Ulnar artery dominance in all digits is uncommon, while complete radial artery dominance has been reported in 28% of hands.^7^ Furthermore, in about 20% of individuals, the ulnar and radial arteries in the hand may be supplemented by a median artery that supplies the superficial palmar arch, and by the anterior interosseous artery, which contributes to the deep palmar arch.^7^ These variations tend to be unilateral.

### Brachial artery

**FIGURE 2:**
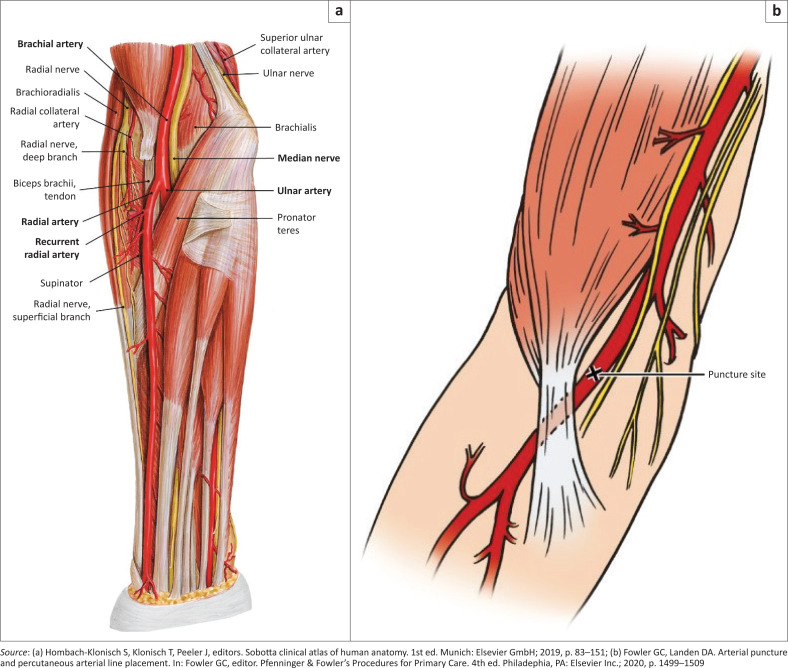
(a) Anatomy of the brachial artery. (b) Puncture site for brachial artery cannulation.

The brachial artery begins as the continuation of the axillary artery, coursing along the medial side of the antecubital fossa just lateral to the median nerve.^8^ It divides at approximately the level of the neck of the radius to become the ulnar and radial arteries (Figure 2a).^8^

The brachial artery is more easily identified when the elbow is fully extended. It can be located by palpating the medial epicondyle of the humerus and moving laterally until the medial edge of the biceps. The arterial pulsation is most easily identified at the level of the proximal flexor crease of the antecubital fossa.^8^ The preferred location for puncture or cannulation of the brachial artery is in, or just proximal to, the antecubital fossa and directly above the brachial artery pulse (Figure 2b).^8,9^

### Femoral artery

**FIGURE 3:**
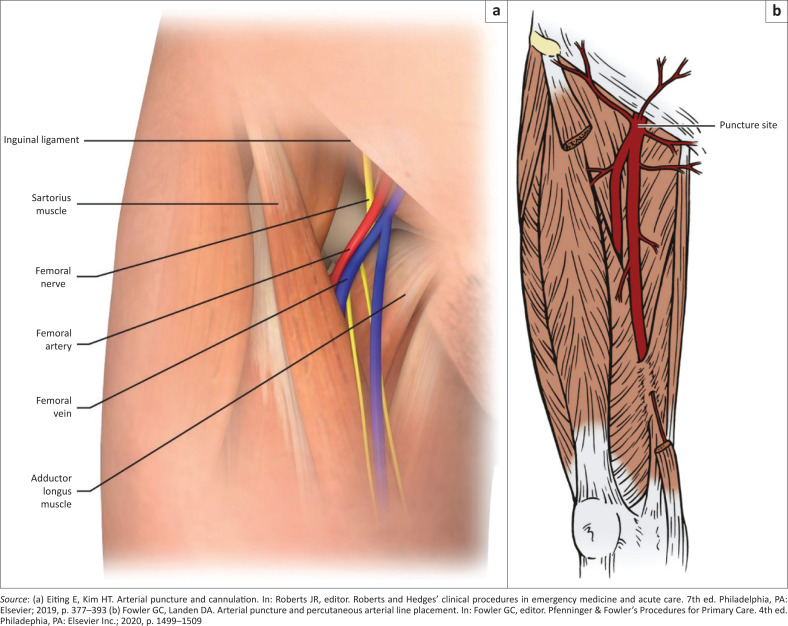
(a) Anatomy of the femoral artery. (b) Puncture site for femoral artery cannulation.

The femoral artery is the direct continuation of the iliac artery and enters the thigh after passing below the inguinal ligament.^10^ It lies approximately midway between the anterior superior iliac spine and the tubercle of the pubic symphysis (Figure 3a).

Femoral arterial puncture must always occur distal to the ligament to prevent uncontrolled haemorrhage into the pelvis or peritoneum. The puncture site should however not be too distal to the inguinal ligament because the femoral artery splits into the superficial femoral and the deep femoral arteries (Figure 3b).^10^

## Patient preparation

The selection of an appropriate insertion site, creation of a sterile field, positioning of the selected site and anaesthesia form part of the preparations for arterial line placement.

### Site selection

The first step in the selection of an arterial line insertion site is the location of a palpable arterial pulse. The peripheral arteries (radial and brachial) are more easily located. They also have lower infection risk compared to the femoral artery.^11^

**FIGURE 4:**
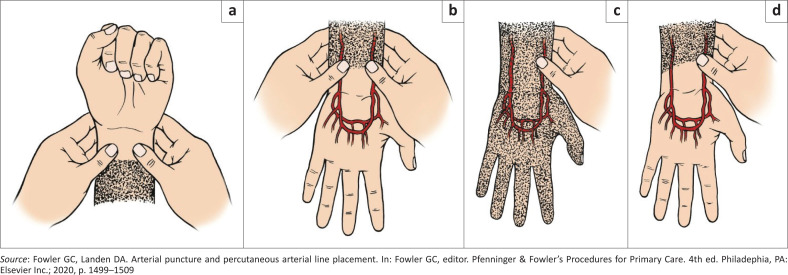
(a–d) The modified Allen test.

The most common site for arterial line insertion is the radial artery.^12,13^ The radial artery site is easily accessible, easy to clean and the most comfortable for the patient.^14^ It is important to check for adequacy of collateral blood flow to the hand from the corresponding ulnar artery before cannulation of a radial artery. The best method for assessing radial artery patency and collateral competency is by colour Doppler ultrasonography.^15^ Where this equipment or expertise is not available, the modified Allen test could be employed (see Figure 4).

The modified Allen test is performed by elevating the hand of interest and having the patient clench his and/or her fist for 30 s (Figure 7). Digital pressure is applied simultaneously over the radial and ulnar arteries of the hand to occlude them until the patient’s hand blanches. The hand is then unclenched and the digital pressure over the ulnar artery is released. The time it takes for the colour to return to the hand is measured in seconds.^12^

The interpretation of the modified Allen test result is not consistent in the literature. The interpretation by Clarke et al.^14^ is as follows:

- Return of colour to the hand within 5 s – 15 s is normal.
- Return of colour to the hand after 15 s is abnormal.

The different interpretations of test result by Eiting et al.,^10^ Grisham et al.,^16^ and Zisquit et al.^17^ reveal the inconsistency in the interpretation of the modified Allen test results.

Besides these inconsistencies, the modified Allen test has also shown significant interobserver variability and lacks predictive accuracy for subsequent hand ischaemia.^11^ Despite the imperfections of the modified Allen test, many experts recommend that the test be performed before radial line insertion and another insertion site be considered if the test is abnormal.^9^ Poor collateral perfusion is present in 12% of people.^18^

Multiple failed attempts at cannulation may lead to vasospasm, making further attempts even more difficult. Such needle-induced vasospasm can be reduced with the following measures:

- Apply pressure over the puncture site to ensure haemostasis and wait several minutes^19^ to allow the artery to recover from spasm.
- Subcutaneous infiltration of lignocaine around the puncture site^12^ or give intravenous midazolam.^20^
- Move to a site proximal to the previous site of puncture.^19^

The femoral artery is the second most common artery used for catheterisation. It is essential to identify the femoral artery below the inguinal ligament in order to avoid repeated punctures of the artery above the ligament. Punctures above the inguinal ligament could lead to significant bleeding from the iliac vessels to the retroperitoneum. The femoral artery gives a pressure that more accurately reflects the pressure in the aorta. This is important in conditions such as septic shock when large doses of vasopressors employed could cause severe vasoconstriction in a peripheral line leading to inaccurate pressure readings. The femoral artery site is, however the most likely to get infected compared to other sites.^14^

Sites with abnormal anatomy should be avoided. A scar in the arterial site may suggest previous surgery or injury, which may have altered the site anatomy. Inserting an arterial line in sites with altered anatomy may be very difficult and may give inaccurate readings.^14^

### Creation of a sterile field

To start with, place sterile equipment on sterile covered equipment tray. For peripheral arterial sites, wear sterile gloves and clean the site area with chlorhexidine antiseptic solution, which is allowed to dry before proper positioning of the site and application of a sterile fenestrated drape. The use of 2% aqueous chlorhexidine-gluconate solution for skin preparation is associated with lower blood stream infection rates than povidone-iodine.^2^ For central arterial sites, full barrier precautions including masks, caps and eye protection can be used to minimise the risk of disease transmission from blood splatter.^11,21^

Every arterial catheterisation carries the risk of eventual local infection, and every local infection carries the risk of septic complications such as arterial catheter-related bloodstream infections.^22^ The most common route of infection is extraluminal.^23^ The lumen of the artery can become locally infected when the skin microflora migrate to the lumen via the catheter, either during the process of insertion or during the time in which the catheter is dwelling within the patient.^22^

### Patient positioning

The ideal position depends on the selected insertion site. For radial artery cannulation, place the forearm in supination and parallel to the floor with the wrist dorsiflexed to 30 ° – 45 ° and supported underneath the dorsal aspect with a sterile gauze roll or towel.^12,14,24^ For femoral artery cannulation, place the patient supine with the hip of the chosen site slightly externally rotated.

### Anaesthesia

Anaesthesia is not required in unresponsive patients. The skin and subcutaneous tissue around the artery should be infiltrated with 1 mL – 2 mL 1% lignocaine solution if the patient is conscious. Maintain gentle negative pressure on the syringe plunger to elicit inadvertent intravascular needle placement and prevent intravascular injection. Avoid to make big skin bleb that obscures palpation of the artery and ensure that the local anaesthetics covers the areas where stitches will be inserted. Do not use lignocaine with epinephrine solution as it can constrict the artery and renders the cannulation process more difficult. Alternative local anaesthetics for arterial line placement include the use of lignocaine-tetracaine patch or lignocaine-prilocaine cream.^12,25^

## Procedure

The most commonly used methods for arterial line placement are the catheter-over needle-technique and the catheter-over-wire technique (the Seldinger and modified Seldinger techniques). The choice of method depends on the selected site for cannulation, available equipment and operator preference. Both methods can be used for radial artery catheterisation but the catheter-over-needle method is more commonly used in infants and neonates, while the catheter-over-wire method is more common in adults and bigger children. For femoral artery cannulation, the catheter over-wire-method is the preferred technique but the catheter-over-needle technique can also be used either alone or in combination with an over-wire technique if there is need for a longer indwelling catheter.^12^

The artery to be cannulated is identified by palpation over anatomic landmarks or ultrasound guidance. It is more effective to first palpate the artery using the landmark technique and then confirm with ultrasound or Doppler ultrasound, which is further used to guide the needle and catheter insertion. Ultrasound guidance is particularly useful to identify and access arteries in patients with small arteries, previous arterial lines or attempts in the same site or hypotension. It can also assist to distinguish veins, which typically collapse under gentle pressure of the probe, from pulsating arteries.^12^ The advantages of ultrasound guidance include higher first-time success rate, reduced number of insertion attempts, shortened cannulation time, reduced complications and ability to identify anatomic variations.^12,26^

**FIGURE 5:**
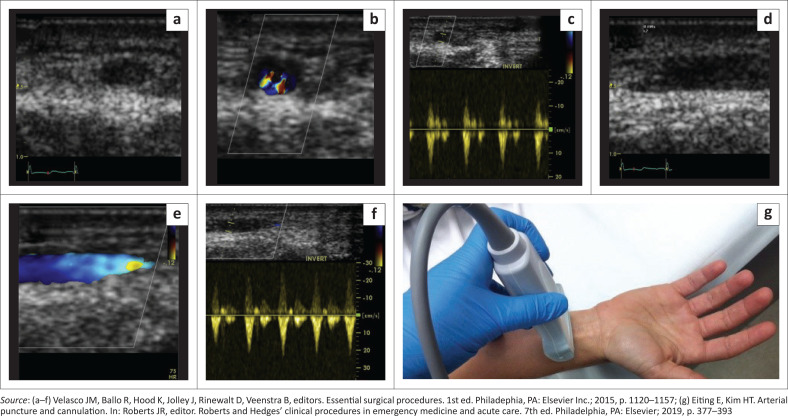
(a–g) Surface ultrasound imaging of the radial artery.

Following the cleaning and preparation of the skin over the artery, a sterile probe cover or a sterile glove over the linear array ultrasound probe is applied (Figure 5g). The target vessel is typically easiest to identify in the transverse (Figure 5a) or longitudinal orientation (Figure 5d), with phasic blood flow (Figure 5b and Figure 5e) as well as pulsatile flow on pulse-wave doppler (Figure 5c and Figure 5f).

### The catheter-over-needle technique

The catheter-over-needle technique is the most basic method for intra-arterial line placement (see Table 1).^12^ Similar to the technique of venous cannula insertion, a needle with an integrated catheter is inserted into the arterial lumen and the catheter is advanced over the needle. This technique is most useful in superficially located arteries such as the radial artery. It is the preferred technique for radial artery catheterisation in neonates and infants who usually have small-diameter arteries that pose difficulties in threading a guide wire into the arterial lumen. The steps for the catheter-over-the needle technique include:^12^

**FIGURE 6:**
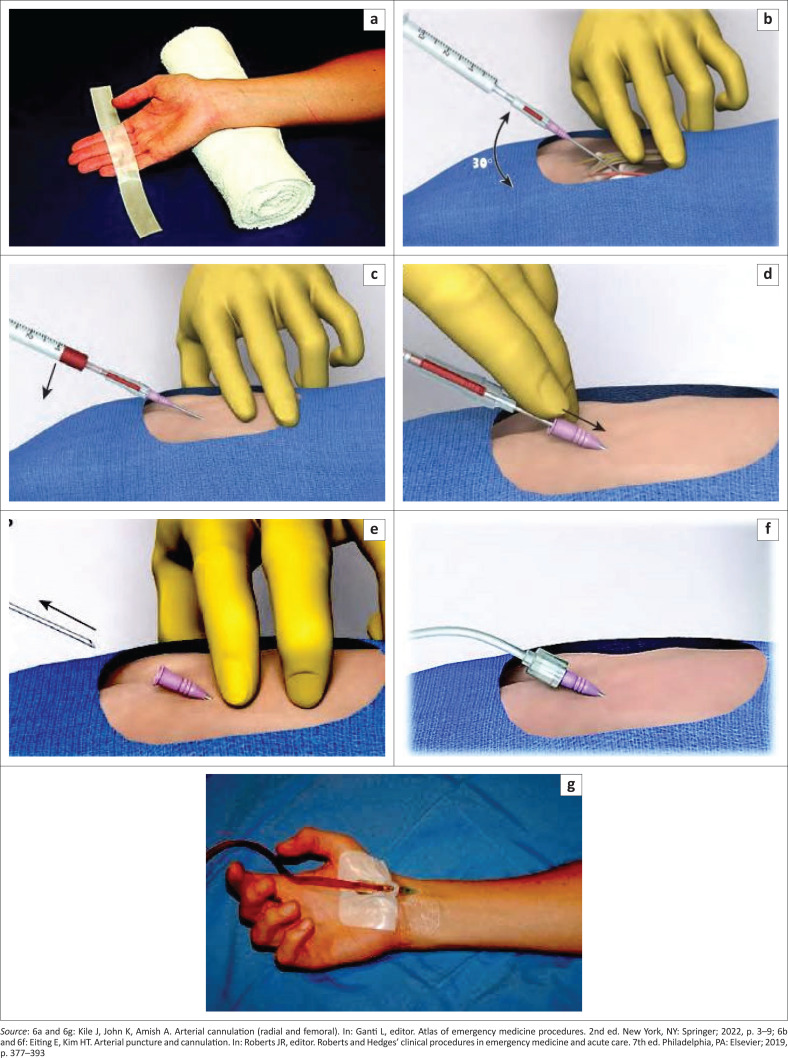
(a–g) Catheter-over-needle technique of arterial cannulation (radial artery).

- Ensure proper positioning of the patient to maximise exposure of the skin overlying the chosen artery. For the radial artery, this is best accomplished by dorsiflexing the wrist and supporting this position with a small towel rolled up under the dorsal wrist surface (Figure 6a).^8^
- Palpate the artery with the second and third digits of the nondominant hand and confirm by visualising the artery with ultrasound device in the nondominant hand if equipment and skill available (see Figure 5).
- Puncture the skin proximal to the palpating fingers over the arterial pulsations with the needle bevel facing up and advancing at an angle of 30 ° – 45 ° towards the pulsation (Figure 6b). If using ultrasound guidance, follow the tip of the ultrasound device with the tip of the needle in the short axis view or preferably use the long axis view, which has the advantage of allowing visualisation of the length of the artery and needle during catheterisation.
- Observe the needle hub for a flash of bright red blood, which signifies arterial puncture (Figure 6c). Lower the needle-catheter unit to an angle of 10 ° – 20 ° and insert the needle 1 mm – 2 mm further to advance the catheter into the arterial lumen. This further needle advancement is necessary to obviate placement of the catheter outside the arterial lumen because the leading needle tip, which caused the initial flash of arterial blood extends beyond the catheter tip by approximately 1 mm – 2 mm.^11^ (Figure 6c)
- Stabilise the needle and advance the catheter over the needle until the catheter hub is at the skin level (Figure 6d).
- Remove the needle and attach the catheter to the arterial line tubing (Figure 6e and Figure 6f).
- Secure the catheter with sutures or adhesive strips and apply sterile dressing over the site (Figure 6g).

**TABLE 1:** Comparison of the catheter-over-needle, Seldinger and modified Seldinger techniques for arterial line insertion.

Criteria	Catheter-over-needle	Seldinger	Modified Seldinger
Equipment^12^	Integrated catheter-needle system	Separate needle, catheter and guidewire components	Integrated needle-catheter-guidewire system
Procedure times^27^	Quick	Quicker	Quickest
Success rate^27^	Good	Best	Better
Number of punctures to succeed^27^	Most number of punctures	Least number of punctures	More number of punctures
Ultrasound guidance used with technique^12,26^	Higher first time success rate Reduced number of punctures or attempts Shortened procedure time Reduced complications	Higher first time success rate Reduced number of punctures or attempts Shortened procedure time Reduced complications	Higher first time success rate Reduced number of punctures or attempts Shortened procedure time Reduced complications

*Source:* Adapted from Ogle S, Kulungowski AM. Arterial line placement medscape [homepage on the Internet]; 2022 [updated 2022 Sep 21; cited 2024 May 15]. Available from: https://emedicine.medscape.com/article/1999586-overview; Kile J, John K, Amish A. Arterial cannulation (radial and femoral). In: Ganti L, editor. Atlas of emergency medicine procedures. 2nd ed. New York, NY: Springer; 2022, p. 3–9; Beards SC, Doedens L, Jackson A, et al. A comparison of arterial lines and insertion techniques in critically ill patients. Anaesthesia. 1994;49(11):968–373. https://doi.org/10.1111/j.1365-2044.1994.tb04316.x

### The catheter-over-wire technique

The catheter-over-wire method includes the Seldinger and modified Seldinger techniques.^26^ The two techniques are similar but the Seldinger technique uses separate equipment components while the modified Seldinger technique uses an integrated needle-catheter-wire system. Both techniques are used for superficial arteries and are preferred for femoral arterial line placements. They are not recommended for routine radial arterial line placements in neonates and infants because of the small diameters of their vessels, which impede the easy advancement of guide wires.

#### The Seldinger technique

The steps for the Seldinger technique include:^12^

**FIGURE 7:**
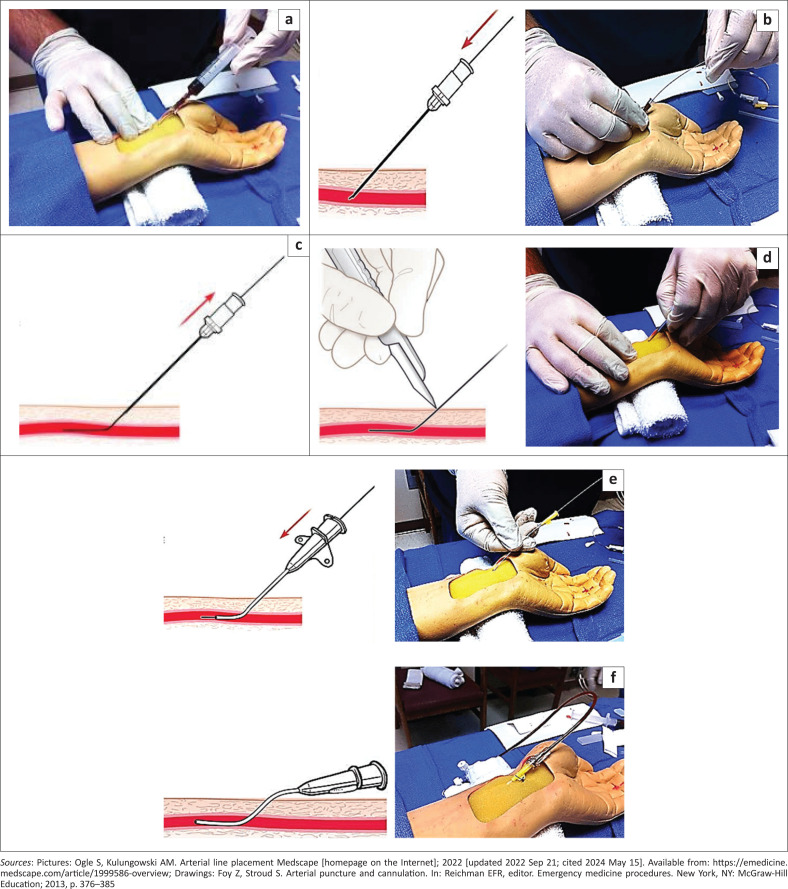
(a–f) The Seldinger technique for arterial cannulation.

- Open the arterial line kit and check the guide wire to ensure that it moves freely through the introducer needle.
- Palpate the artery with the second and third digits of the nondominant hand.
- Properly position the patient as previously described.
- Attach the needle to the syringe and puncture the skin proximal to your fingers over the arterial pulsation with the needle bevel facing up and advancing at an angle of 30 ° – 45 ° towards the pulsation (Figure 7a).
- Advance the needle with slight negative pressure in the syringe until free return of blood is visualised (Figure 7a).
- Remove the syringe and advance the guide wire into the artery (Figure 7b and Figure 7c).
- Make a small incision (not stab incision) next to the skin puncture site of the guidewire using a small scalpel blade to facilitate inserting the catheter (Figure 7d).^8^ Face the sharp edge of the scalpel away from the guide wire, to avoid severing the wire.^26^
- Remove the needle while holding the guide wire in place and then advance the catheter over the guide wire into the artery. The return of pulsatile blood from the catheter hub confirms proper placement of the catheter (Figure 7e).
- Attach the catheter to the arterial line tubing (Figure 7f).
- Secure the catheter with sutures or adhesive strips and apply sterile dressing over the site.

#### The modified Seldinger technique

The steps for the modified Seldinger technique include:^12^

**FIGURE 8:**
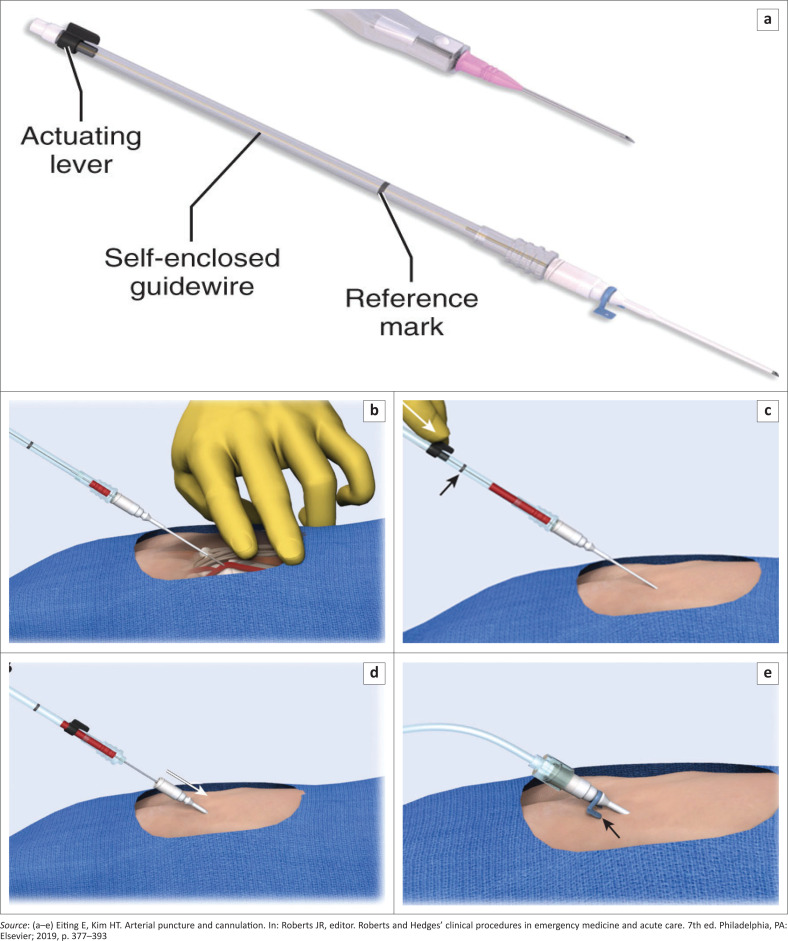
(a–e) Arrow arterial catheterization kit.

- Open the arterial line kit, assemble the catheter and guide wire, ensure that the guide wire moves freely and then confirm that the guide wire is fully retracted (Figure 8a).
- Palpate the artery with the second and third digits of the non-dominant hand.
- Puncture the skin proximal to your fingers over the arterial pulsation with the needle bevel facing up and advancing at an angle of 30° – 45° towards the pulsation.
- Advance the needle until a flash of blood appears in the hub (Figure 8b).
- Stabilise the needle and advance the guide wire into the artery by moving the actuating lever as far forward as possible (Figure 8c).
- Grasp the catheter hub and advance the catheter over the needle and guide wire into the artery (Figure 8d). While holding the catheter hub in place, withdraw the needle and guide wire as a single unit. The return of pulsatile blood from the catheter hub confirms proper placement of the catheter.
- Attach the catheter to the arterial line tubing (Figure 8e).
- Secure the catheter with sutures or adhesive strips and apply sterile dressing over the site.

## Complications

The most common complications with arterial line insertion are arterial occlusion, bleeding from the puncture site, and haematoma.^1,28^ The less common complications include infection (localised catheter-site infection, suppurative thromboarteritis, catheter-related blood stream infection), distal ischaemia and necrosis, vasospasm, thromboembolism, pseudoaneurysm formation, arteriovenous fistula, air embolism, compartment syndrome, carpal tunnel syndrome, and nerve injury.

Clinically significant complications occur in less than 1% of arterial line placements.^1,28^ The risk of these complications can be minimised with appropriate knowledge of the relevant anatomy and procedural skills.^12^ Table 2 shows the NHS Lothian Mastery Skills Pathway suggested strategies that could be adopted to minimise some of these complications.^14^

**TABLE 2:** Strategies to minimise risk of complications from arterial line insertion.

Complication	Strategy to minimise risk
**Thromboembolic**	
Air embolism	Prime the transducer adequately to ensure there is no air in the system prior to connecting to arterial line
Wire embolism	Ensure wire is controlled throughout the procedure, aiming to have a grip on it as much as possible
Catheter-related thrombosis	Appropriate venous thromboembolism (VTE) thromboprophylaxis
**Mechanical**	
Haemorrhage	Optimise coagulopathy prior to insertion
Nerve and/or vein injury	Careful palpation prior to insertion or use of ultrasound guidance
Artery injury (vasospasm, dissection, pseudoaneurysm)	Avoid repeated attempts, rest artery or attempt at alternative site if injury suspected, avoid end artery insertion if possible.
Haematoma formation	Avoid multiple attempted insertions at the same site, correct coagulopathy prior to attempt, apply adequate pressure (force and time) over site if bleeding
**Infectious**	
Catheter colonisation	Aseptic technique on insertion. Use of 2% chloraprep to clean skin for insertion.
Catheter-related blood stream infection	Transparent dressing. Daily review of need for arterial line. Remove if signs of infection.
**Ongoing line use**	
Disconnection and exsanguination	Monitoring of line in critical care setting, always transduced
Intra-arterial injection	All accessible ports clearly labelled

*Source*: From Clarke B, Monro-Sommerville T, Edgar S, et al. Arterial line insertion. Mastery Skills Pathway [homepage on the Internet]. [cited 2024 May 15]. Available from: http://www.scotlanddeanery.nhs.scot>media>arterial_line_insertion.pdf

## Aftercare

**FIGURE 9:**
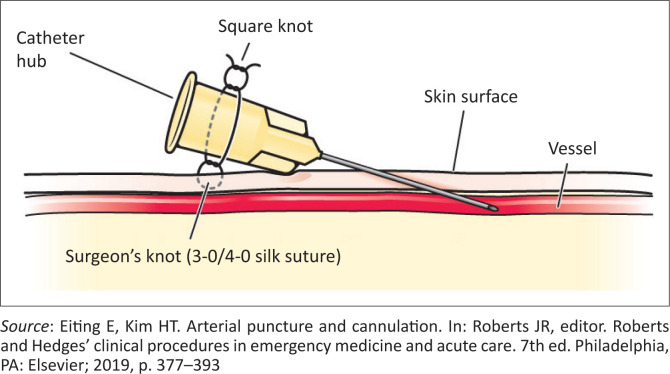
Securing a vascular catheter to adjacent skin.

The following are important care points for the patient after the insertion of an arterial line:

- Maintain firm pressure for 10 min or longer after removing a peripheral artery catheter and longer after femoral cannulation or if the patient has received anticoagulants. Five minutes of pressure is sufficient after puncture for a blood gas sample in an individual with normal coagulation.^10^
- An arterial catheter must be secured to the skin to prevent inadvertent dislodgement, haematoma formation, and exsanguination.^4^ Silk (2–0) or nylon (4–0) provides good anchoring,^10^ as shown in Figure 9. It is important to avoid pinching the skin too tightly. Further secure the catheter and its connecting tubing with sterile sponges and adhesive tape.^10^
- A splinting device should be used to secure the limb in the desired position for optimal monitoring if the catheter is in the wrist, arm, or foot.^8^
- Assess the extremity distal to the catheter for evidence of ischaemia.
- Flush the arterial catheter with sterile saline solution. A three-way stopcock can be interposed between the patient and the transducer for blood gas sampling and to allow flushing of the system. Flushing can be periodic or continuous. The use of heparinised saline has not been shown to improve functionality, prevent thrombotic complications, or increase the duration of catheter patency when compared to saline.^8,10^

## Indications for removal of arterial line

The continued need for an indwelling arterial line should be evaluated daily and should be removed in any of the following conditions^29^:

- Arterial blood pressure monitoring no longer necessary.
- Frequent arterial blood sampling no longer needed.
- Infection/sepsis noted.
- Bleeding from the site/haematoma formation.
- Neurovascular compromise.
- Arterial line system failure (e.g. kinked catheter, thrombus at the tip).

### Procedure for removal of an arterial line

It is important to do coagulation studies (international normalised ratio [INR], partial thromboplastin time [PTT] and platelet count) before removing an arterial line. The removal of an arterial line is an aseptic procedure that requires hand washing, use of hand gloves, protective gown, face mask with shield regardless of catheter location.^29^ The site is cleaned with antiseptic solution and the sutures or adhesive securement device is removed. A sterile dressing is used to apply pressure over the arterial puncture site. The catheter is then slowly pulled out whilemaintaining pressure over the artery and skin puncture site. The applied pressure should continue for 5 min – 10 min after removal of the catheter from the radial and femoral arteries. The removed catheter should be inspected to ensure that it is intact to avoid particulate embolism.^10,29^

After catheter removal the pulses at the puncture site and the distal part of the limb should be checked for signs of haematoma and distal ischaemia.^29^ The check for these complications should be performed every 5 min for the initial period of 30 min, then continued every 30 min for 1 h, and then hourly for 4 h.^29,30^

## Conclusion

This article has provided information to the clinician about arterial line insertion. Indications, contra indications, anatomical landmarks, complications, and the equipment required is outlined in this article.

## References

[1] Hager HH, Bracken burns. Artery cannulation. Treasure Island, FL: StatPearls Publishing; 2024.

[2] Hanson C. Procedures in critical care. Chapter 35. Arterial line cannulation [homepage on the Internet]. The McGraw-Hill Company; 2009 [cited 2024 Mar 05]. Available from: https://accessanesthesiology.mhmedical.com/content.aspx?bookid=414sectionid=41840262

[3] Abdel-Kader A, Kaushal N, Shah R, et al. A novel technique to maintain radial arterial Catheter position: The arterial Catheter stabilizer. Open J Anesthesiol. 2016;6(12):193–197. 10.4236/ojanes.2016.612029

[4] Gibbons RC. Arterial line placement. In: Campo TM, Lafferty KA, editors. Essential procedures for emergency, urgent, and primary care settings: A clinical companion. 3rd ed. New York, NY: Springer Publishing Company; 2022, p. 251–261.

[5] Yalcin B, Kocabiyik N, Yazar F, Kirici Y, Ozan H. Arterial variations of the upper extremities. Anatom Sci Int. 2006;81(1):62–64. 10.1111/j.1447-073X.2006.00110.x 16526599

[6] Nguyen JD, Black AC, Duong H. Anatomy, shoulder and upper limb, hand arteries [homepage on the Internet]. StatPearls Publishing LLC; 2024 [cited 2024 May 03]. Available from: https://www.ncbi.nlm.nih.gov/books/NBK546583/31536192

[7] Standring S. Gray’s anatomy: The anatomical basis of clinical practice. 42th ed. Amsterdam: Elsevier Health Sciences; 2016.

[8] Foy Z, Stroud S. Arterial puncture and cannulation. In: Reichman EFR, editor. Emergency medicine procedures. New York, NY: McGraw-Hill Education; 2013, p. 376–385.

[9] O’Connell C. Arterial puncture. In: Dehn R, Asprey D, editors. Essential clinical procedures. 4th ed. Expert Consult-Online and Print: New York, NY: Elsevier Health Sciences; 2021.

[10] Eiting E, Kim HT. Arterial puncture and cannulation. In: Roberts JR, editor. Roberts and Hedges’ clinical procedures in emergency medicine and acute care. 7th ed. Philadelphia, PA: Elsevier; 2019, p. 377–393.

[11] Theodore AC, Clermont, Dalton A. Intra-arterial catheterization for invasive monitoring: Indications, insertion techniques, and interpretation [homepage on the Internet]. UpToDate; 2024 [cited 2024 Mar 05]. Available from https://www.uptodate.com/contents/intra-arterial-catheterization-for-invasive-monitoring-indications-insertion-techniques-and-interpretation

[12] Ogle S, Kulungowski AM. Arterial line placement medscape [homepage on the Internet]; 2022 [updated 2022 Sep 21; cited 2024 May 15]. Available from: https://emedicine.medscape.com/article/1999586-overview.

[13] Gu WJ, Wu XD, Wang F, et al. Ultrasound guidance facilitates radial artery catheterization: A meta-analysis with trial sequential analysis of randomized control trials. Chest. 2016;149(1):166–179. 10.1378/chest.15-1784 26426094

[14] Clarke B, Monro-Sommerville T, Edgar S, et al. Arterial line insertion. Mastery Skills Pathway [homepage on the Internet]. [cited 2024 May 15]. Available from: https://www.scotlanddeanery.nhs.scot>media>arterial_line_insertion.pdf

[15] Jarvis MA, Jarvis CL, Jones PR, et al. Reliability of Allen’s test in selection of patients for radial artery harvest. Ann Thorac Surg. 2000;70(4):1362–1365. 10.1016/S0003-4975(00)01551-4 11081899

[16] Grisham J, Stankus JL. Arterial puncture and cannulation. Reichman’s Emergency Medicine procedures. 3rd ed. New York, NY: McGraw-Hill; 2019, p. 623–634.

[17] Zisquit J, Velasquez J, Nedeff N. Allen test. Continuing Education Activity [homepage on the Internet]. StatPearls Publishing; 2024 [updated 2022 Sep 19; cited 2024 May 11]. Available from: https://www.ncbi.nlm.nih.gov/books/NBK507816/29939593

[18] Hignett R, Stephens R. Radial arterial lines. BJ Hosp Med. 2006;65(5):3–5. 10.12968/hmed.2006.67.Sup5.21077 16729631

[19] Tam M, Roberts TJ, Erickson MF. Radial artery sheath insertion [homepage on the Internet]. Medscape; 2024 [updated 2024 May 03; cited 2024 Jun 03]. Available from: https://emedicine.medscape.com/article/2036480-overview

[20] Cho SA, Jang YE, Ji SH, et al. Ultrasound-guided arterial catheterization. Anesth Pain Med (Seoul). 2021;16(2):119–132. 10.17085/apm.21012 33866769 PMC8107253

[21] O’Grady NP, Alexander M, Burns LA, et al. Guidelines for the prevention of intravascular catheter-related infections. Am J Infect Control. 2011;39(4 Suppl 1):S1–S34. 10.1016/j.ajic.2011.01.003 21511081

[22] Hambsch ZJ, Kerfeld MJ, Kirkpatrick DR, et al. Arterial catheterization and infection: Toll-like receptors in defense against microorganisms and therapeutic implications. Clin Transl Sci. 2015;8(6):857–870. 10.1111/cts.12320 26271949 PMC4703511

[23] Safdar N, O’Horo JC, Maki DG. Arterial catheter-related bloodstream infection: Incidence, pathogenesis, risk factors and prevention. J Hosp Infect. 2013;85(3):189–195. 10.1016/j.jhin.2013.06.018 24070632

[24] Ramsey L. Arterial line placement: Basics for medical students [homepage on the Internet]. EMResident. 2018 [cited 2024 Jun 01]. Available from: https://www.emra.org/emresident/article/arterial-line-placement

[25] UCSF Standardized procedure. Arterial catheter insertion (adult) [homepage on the Internet]. [cited 2024 Jun 17]. Available from: https://medicalaffairs.ucsf.edu/standardized-procedures

[26] Kile J, John K, Amish A. Arterial cannulation (radial and femoral). In: Ganti L, editor. Atlas of emergency medicine procedures. 2nd ed. New York, NY: Springer; 2022, p. 3–9.

[27] Beards SC, Doedens L, Jackson A, et al. A comparison of arterial lines and insertion techniques in critically ill patients. Anaesthesia. 1994;49(11):968–373. 10.1111/j.1365-2044.1994.tb04316.x 7802244

[28] Nutall G, Burckhardt J, Hadley A, et al. Surgical and patient risk factors for severe arterial line complications in adults. Anesthesiology. 2016;12(3):590–597. 10.1097/ALN.0000000000000967 26640979

[29] Wang H, He L, Han C, et al. Evidence-based systematic review of removal of peripheral arterial catheter in critically ill adult patients. BMC Anesthesiol. 2024;24:79. 10.1186/s12871-024-02458-0 38408893 PMC10895724

[30] LHSC Procedure for central line management [homepage on the Internet]. [cited 2024 Jun 26]. Available from: https://www.lhsc.on.ca/critical-care-trauma-centre/procedure-removal-of-peripheral-arterial-line

